# Activation of the PI3K/mTOR Pathway following PARP Inhibition in Small Cell Lung Cancer

**DOI:** 10.1371/journal.pone.0152584

**Published:** 2016-04-07

**Authors:** Robert J. Cardnell, Ying Feng, Seema Mukherjee, Lixia Diao, Pan Tong, C. Allison Stewart, Fatemeh Masrorpour, YouHong Fan, Monique Nilsson, Yuqiao Shen, John V. Heymach, Jing Wang, Lauren A. Byers

**Affiliations:** 1 Department of Thoracic/Head and Neck Medical Oncology, The University of Texas MD Anderson Cancer Center, Houston, Texas, United States of America; 2 Biomarin Pharmaceutical Inc., Novato, California, United States of America; 3 Departmant of Bioinformatics and Computational Biology, The University of Texas MD Anderson Cancer Center, Houston, Texas, United States of America; University of Navarra, SPAIN

## Abstract

Small cell lung cancer (SCLC) is an aggressive malignancy with limited treatment options. We previously found that PARP is overexpressed in SCLC and that targeting PARP reduces cell line and tumor growth in preclinical models. However, SCLC cell lines with PI3K/mTOR pathway activation were relatively less sensitive to PARP inhibition. In this study, we investigated the proteomic changes in PI3K/mTOR and other pathways that occur following PAPR inhibition and/or knockdown *in vitro* and *in vivo*. Using reverse-phase protein array, we found the proteins most significantly upregulated following treatment with the PARP inhibitors olaparib and rucaparib were in the PI3K/mTOR pathway (p-mTOR, p-AKT, and pS6) (p≤0.02). Furthermore, amongst the most significantly down-regulated proteins were LKB1 and its targets AMPK and TSC, which negatively regulate the PI3K pathway (p≤0.042). Following PARP knockdown in cell lines, phosphorylated mTOR, AKT and S6 were elevated and LKB1 signaling was diminished. Global ATP concentrations increased following PARP inhibition (p≤0.02) leading us to hypothesize that the observed increased PI3K/mTOR pathway activation following PARP inhibition results from decreased ATP usage and a subsequent decrease in stress response signaling via LKB1. Based on these results, we then investigated whether co-targeting with a PARP and PI3K inhibitor (BKM-120) would work better than either single agent alone. A majority of SCLC cell lines were sensitive to BKM-120 at clinically achievable doses, and cMYC expression was the strongest biomarker of response. At clinically achievable doses of talazoparib (the most potent PARP inhibitor in SCLC clinical testing) and BKM-120, an additive effect was observed *in vitro*. When tested in two SCLC animal models, a greater than additive interaction was seen (p≤0.008). The data presented here suggest that combining PARP and PI3K inhibitors enhances the effect of either agent alone in preclinical models of SCLC, warranting further investigation of such combinations in SCLC patients.

## Introduction

Small cell lung cancer (SCLC) is the most aggressive form of lung cancer, with a 5-year survival rate of only 6% [1]. There are approximately 30,000 deaths from SCLC annually in the United States (which makes up 13% of lung cancers), making SCLC alone the 8^th^ leading cause of cancer death in the US in 2011 [2, 3]. Most cases of SCLC respond to chemotherapy and radiation initially. However, relapse is nearly universal, and in a majority of patients further systemic therapy produces no response. Unlike non–small cell lung cancers (NSCLC), SCLC currently has no approved targeted therapies with demonstrated benefit for patients. Therefore, the development of new, active, and potentially targeted drugs for the treatment of SCLC represents a major unmet medical need.

We previously reported that poly-ADP ribose polymerase 1 (PARP1) is overexpressed in SCLC, identifying PARP as a potential therapeutic target in this cancer [4]. Further work by our group has shown that PARP1/2 inhibitors talazoparib (BMN 673), olaparib (AZD2281), and rucaparib (CO-338, AG 014699) have single-agent activity in preclinical models [4, 5]. While PARP inhibitors are in late stage development for the treatment of ovarian cancers (olaparib approved, rucaparib Phase 3), based on this preclinical data, several clinical studies of PARP inhibitors are being conducted in SCLC to investigate the effect of these drugs as single agents or in combination with chemotherapy (Phase I–talazoparib, Phase II olaparib and veliparib) [6]. In the Phase I study of single-agent talazoparib, we observed partial responses in a subset of patients with relapsed SCLC [7]. However, as with other targeted drugs, identifying mechanisms associated with innate and acquired drug resistance and rational combinations to increase response rates and/or duration is critical for optimizing the clinical application of these drugs [7, 8].

In previous pre-clinical work, using a panel of 8 SCLC cell lines we correlated cell line sensitivity to PARP inhibition with each cell line’s proteomic profile [5]. Cell lines with the greatest activation of the PI3K/mTOR pathway were the least sensitive to talazoparib; with phosphorylated (p)-AKT (T308) and p-AKT(S473) as the top markers of resistance [5]. Genetic alterations in the PI3K/mTOR pathway are not uncommon in SCLC [9, 10], with typically mutually exclusive alterations in *PIK3CA*, *PTEN*, *AKT2*, *AKT3*, *RICTOR*, or *MTOR* found in 36% of patients [10]. Preclinical reports have shown PI3K inhibitors such as PIK75 and PF-4989216 to have activity in SCLC models with *PIK3CA* mutations, but not *PTEN* deficiency, indicating a possible role for PI3K/mTOR-targeted therapy in SCLC [11, 12]. Related to this finding, previous reports in breast cancer have shown that treatment with a PI3K inhibitor delayed tumor growth but increased indicators of DNA damage such as poly-ADP ribose (PAR) [13, 14]. While PARP inhibition alone in these breast cancer models only moderately attenuated growth, the combination of PARP and PI3K inhibition was particularly potent in suppressing growth [13, 14].

As proteomic analysis revealed an inverse correlation between activity of the PI3K/mTOR pathway and response to talazoparib *in vitro* [5], we hypothesized that the addition of PI3K/mTOR inhibition might further sensitize SCLC to PARP inhibitors. We first investigated in SCLC cell lines the intracellular response to PARP inhibition, observing increased PI3K/mTOR signaling following PARP inhibition.

In this study we show for the first time that PI3K/mTOR signaling increases following inhibition of PARP in SCLC and that this may be driven through a reduction in liver kinase B1 (LKB1) signaling–changes validated by PARP1 knockdown. Consequently, we investigated the antitumor effects of combining a PARP inhibitor with a PI3K-specific inhibitor in preclinical models of SCLC. Combination studies targeting PARP and PI3K *in vitro* revealed an additive interaction between these two inhibitors in proliferation assays. Animal studies revealed that this combination has greater effect than either drug alone in reducing tumor volume, providing a strong rationale for the advancement of this combination into clinical studies in SCLC patients.

## Materials and Methods

### Cell lines

Human SCLC cell lines COR-L88, DMS1114, DMS 153, DMS 53, DMS 79, H1048, H1092, H1105, H128, H1341, H1417, H1436, H146, H1672, H1836, H187, H1876, H1930, H196, H1963, H2081, H209, H211, H2141, H2171, H2195, H2227, H2330, H250, H345, H378, H446, H510, H524, H526, H69, H719, H748, H774, H82, H841, H847, H865, H889, and SHP-77 were obtained from ATCC (Manassas, VA) or Sigma-Aldrich (St. Louis, MO); GEMM-derived cell lines Kp1, Kp3, Kp11, and Kp12 [15] and human patient-derived xenograft (PDX) derived cell line NJH29 were all generously provided by Dr. Julien Sage (Stanford University, Stanford CA). All cells were grown in suggested medium supplemented with fetal bovine serum and penicillin/streptomycin. Cells were passaged for fewer than 6 months following receipt.

### Protein analysis

For RPPA and western blot analysis, cells were treated in duplicate with 1μM olaparib (Selleck Chemicals, Houston TX), rucaparib (Selleck Chemicals, Houston TX), or talazoparib (Biomarin Pharmaceutical Inc,Novato CA). Western blots were probed for PARP1 (cs9542), mTOR pS2448 (cs2971), mTOR (cs2983), AKT pT308 (cs9271), AKT (cs9272) S6 pS240,244 (cs2215), S6 (cs2217), LKB1 (cs3050), AMPKα pT172 (cs2532), AMPKα (cs2532) (Cell Signaling Technlogy, Danvers MA), and actin (sc1616, Santa Cruz Biotechnology, Dallas TX).

### Reverse phase protein array

Protein lysates were collected in a buffer containing 1% Triton X-100, 50 mmol/L HEPES (pH 7.4), 150 mmol/L NaCl, 1.5 mmol/L MgCl_2_, 1 mmol/L EGTA, 100 mmol/L NaF, 10 mmol/L NaPPi, 10% glycerol, 1 mmol/L PMSF, 1 mmol/L Na_3_VO_4_, and 10 mg/mL aprotinin. Samples were quantified and protein arrays were printed from lysates and stained as previously described [4, 16]. Briefly, the slide images were quantified by using MicroVigene 4.0 (VigeneTech, Carlisle, MA). The spot level raw data were processed by using the R package SuperCurve [17–19], which returns the estimated protein concentration (raw concentration) and a quality control (QC) score for each slide. Only slides with a QC score >0.8 were used for downstream analysis. The raw concentration data were normalized by median-centering each sample across all the proteins to correct loading bias.

### Proliferation assays

Cells were seeded in 96-well plates at 2,000 cells per well in triplicate for each cell line. After 24 hours, the cells in each well were treated for 24 hours with a PARP inhibitor (talazoparib) and/or PI3K inhibitor (BKM-120, Selleck Chemicals, Houston TX) or with vehicle control. Four days later, proliferation was assayed by Cell Titer Glo (Promega, Fitchburg, WI). For single-drug treatments, median inhibitory concentration (IC_50_) values were estimated by the drexplorer software [20]. Specifically, for each drug combination (at each dose level), the observed (or experimental) effect of the combination was compared to the predicted additive effect. Data was subsequently presented as a percentage of the experimental effect relative to the predicted additive effect (1.1 = +10%; 1 = 0%; 0.9 = -10%). For example, if drug A reduces relative proliferation to 0.8 and drug B to 0.7, then the predicted additive effect would be 1-((1–0.8)+(1–0.7)) = 0.5. If the observed (experimental) effect of the combination on relative proliferation is then 0.3, then the observed effect is greater than the predicted effect of the combination [(1–0.3)/(1–0.5) = 1.4]. Using 10% above or below the predicted additive effect as a cut-off, we then assigned the following groups: Observed/predicted>1.1 = greater than additive; Observed/predicted<0.9 = less than additive; Observed/predicted ≤1.1 and ≥0.9 = additive. For drug combinations, the MacSynergy II approach [21] based on the Bliss Independence model [22] was implemented to calculate various metrics, including synergistic volume, antagonistic volume, overall volume, and extra kill percentage [23].

### Gene knockdown

For stable protein knockdown, lentiviral particles encoding two different short-hairpin RNAs (shRNAs) targeting PARP1 and scramble control shRNA (pGIPZ lentiviral vector, Open Biosystems a division of Dharmacon/GE Healthcare, Lafayette CO) were selected and designated as PARP1-shRNA-1, PARP1-shRNA2, and SCR-shRNA, respectively. The PARP1 shRNA sequences were as follows: *PARP1*-shRNA1: 5′-TAGTTGAACACACTTTCTT-3′; PARP1-shRNA2: 5′-TGATGTTCCAGATCAGGTC-3′. The *STK11* (LKB1) shRNA sequences were as follows LKB1-shRNA1: 5'—GCATTAAAGCAGCGTATC—3'; LKB1-shRNA2: 5’ ATTTATTGCCAAATTTGGG-3’. The shRNA lentiviral particles were incubated with target cells for 24 hours, and cells then were selected in appropriate culture medium containing puromycin (2 μg/mL) for 3 weeks. The resistant colonies were expanded, and knockdown efficiency was validated by qRT-PCR and western blotting.

### ATP quantification

To evaluate the availability of ATP, global ATP concentrations were measured in cells treated or not treated with a PARP inhibitor. ATP concentrations were assayed by the ATPlite Luminescence System per the manufacturer’s instructions (PerkinElmer, Inc., Waltham MA).

### Poly ADP-Ribose (PAR) assay

To evaluate the effect of PARP/PI3K inhibition on PARP1 activity *in vivo*, lysates were prepared from xenografts 2 hours after drug delivery on day 3 of treatment. Xenografts were produced by the method described in the next subsection. PAR levels were assayed by ELISA per the manufacturer’s instructions (Trevigen, Inc., Gaithersburg, MD).

### Animal models

Female Balb/c nude mice were obtained from Shanghai Lingchang BioTechnology Co. LTD (Shanghai, China) or Harlan Laboratories (Houston, TX). This study was carried out in strict accordance with the recommendations in the Guide for the Care and Use of Laboratory Animals of the National Institutes of Health. All the procedures related to animal handling, care and the treatment in this study were approved by the Institutional Animal Care and Use Committee (IACUC) of Shanghai Chempartner (protocol number A998HL0001) or by the University of Texas MD Anderson Cancer Center IACUC (protocol number RM00001191-RN01). All efforts were made to minimize animal suffering. Human NCI-H209 SCLC cells (5×10^6^ cells) or NCI-H1048 tumor cells (3×10^6^ cells) in 0.2 mL of a 1:1 mixture of medium and matrigel were injected subcutaneously into the right flank of each mouse (36 animals per cell line). When tumors reached ~150 mm^3^ average volume, animals (6 per group) were treated orally with vehicle, talazoparib (0.25 mg/kg), or BKM-120 (25 mg/kg) daily for 28 days. Tumor volume and animal weight were measured every 2 to 3 days. A further 3 animals for each group were treated for 3 days; xenograft tumors were harvested from and snap frozen 2–3 hours post treatment on day 3 for further analysis. Treatment efficacy was determined by comparing the tumor volume from drug-treated mice (ΔT) with that from mice treated with vehicle (ΔC) using the ratio (ΔT/ΔC) at day 19 for NCI-H1048 and day 25 for NCI-H209 [24] (last measurement of vehicle treated tumors).

## Results

### PARP inhibition *in vitro* increases PI3K signaling in SCLC

**Fig 1:**
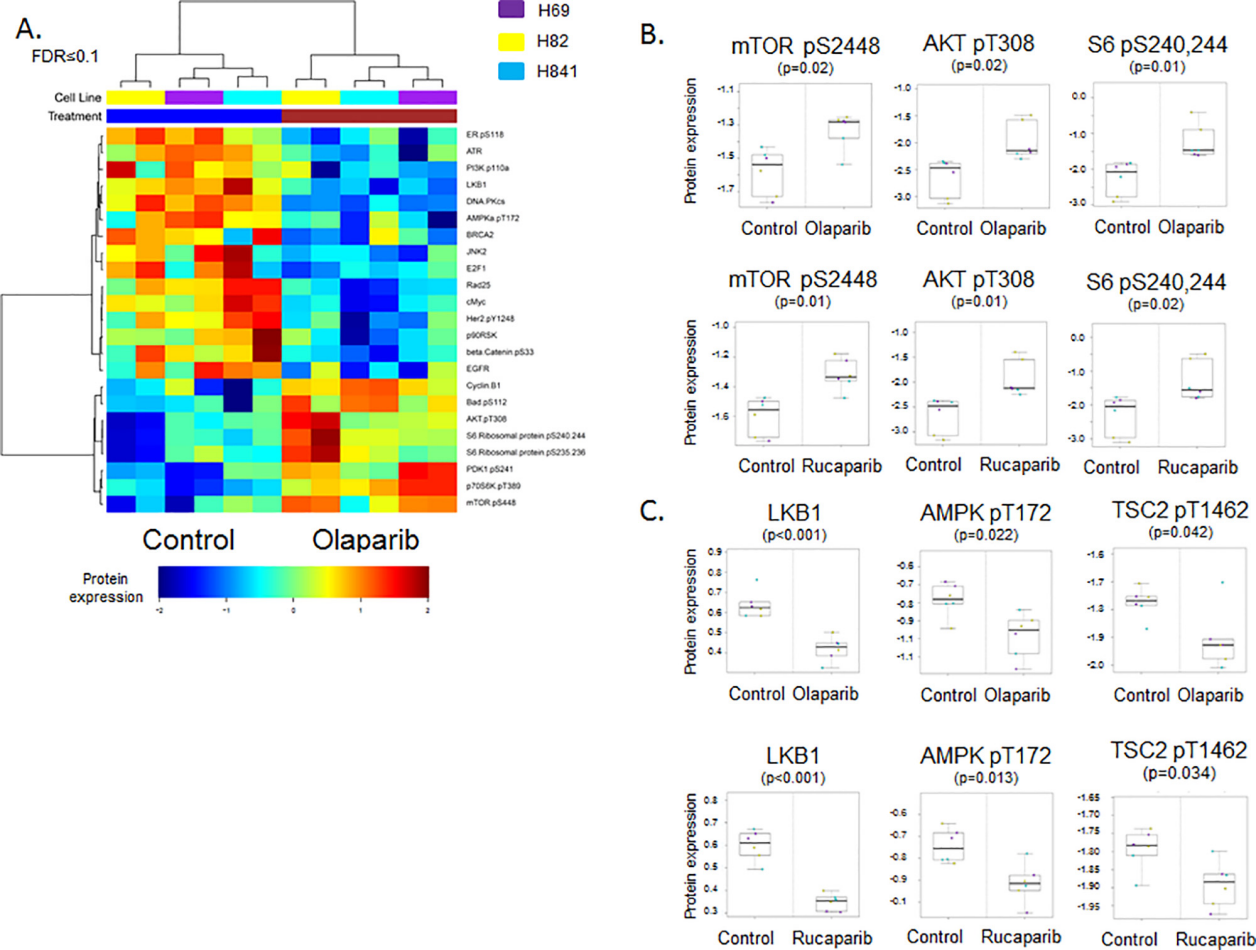
PARP inhibition increases PI3K/mTOR pathway activity through LKB1 inactivation *in vitro*. (A) Hierarchical clustering of proteins identified in lysates from SCLC cell lines (H69, H82, H841) treated with vehicle or olaparib for 24 hours revealed increased activity of the PI3K/mTOR pathway following PARP inhibition (FDR≤0.1, equivalent p-value≤0.037). (B) Individual phosphorylated proteins in the PI3K/mTOR pathway (p-mTor, p-AKT and p-S6 kinase) are increased following treatment with either olaparib or rucaparib (p≤0.02). (C) Lysates from cell lines treated with olaparib or rucaparib versus vehicle for 24 hours show inactivation of the LKB1 pathway (LKB1, pAMPKα, and pTSC2; p≤0.042).

To identify pathways modulated by PARP inhibition, we performed reverse phase protein array (RPPA) which measured changes in 137 total and phosphorylated proteins in key oncogenic pathways including PI3K, MEK and DNA repair in a panel of SCLC cell lines treated with either vehicle or one the PARP inhibitors olaparib (AZD2281), rucaparib (CO-338, AG 014699), or talazoparib (BMN 673) for 24 hours. Analysis of cell lysates collected pre- and post-treatment showed that phosphorylation (activation) of proteins in the PI3K/mTOR pathway was increased in treated cell lines. RPPA analysis revealed that, of 137 total and phosphorylated proteins measured, six of the eight proteins that were most significantly upregulated in response to PARP inhibition (FDR ≤0.1) were in the PI3K/mTOR pathway (including p-mTOR, p-AKT, and p-S6 [p≤0.02] (Fig 1A and 1B) total protein levels were unchanged by RPPA (p≥0.27)). Talazoparib, which has an approximately 10-fold lower IC_50_ in SCLC than olaparib or rucaparib, induced a similar response in H69 cells, as exhibited by increased p-AKT T673 and p-S6 S240,244 following treatment (S1 Fig).

### PARP inhibition drives PI3K/mTOR activation through ATP/LKB1

Our previous studies showed that in addition to PARP, SCLC cell lines also overexpress liver kinase B1 (LKB1) [4]. The LBK1 pathway is a stress-response mechanism that is activated in response to metabolic stresses such as ATP depletion to protect the cell [25]. One consequence of LKB1 activity is suppression of the mTOR pathway [25]. RPPA analysis of SCLC cells treated with olaparib revealed a decrease in activity of the LKB1 pathway (Fig 1A). Further analysis (Fig 1C) showed that treatment with either olaparib or rucaparib significantly reduced LKB1 expression (p<0.001). Phosphorylation of LKB1’s downstream targets, pAMPKα and pTSC2, was also significantly reduced following treatment with PARP inhibitors (p = 0.022 and p = 0.042 with olaparib; p = 0.013 and p = 0.034 with rucaparib for pAMPKα and pTSC2, respectively; total AMPK and TSC protein levels were unchanged as measured by RPPA, p≥0.81).

**Fig 2:**
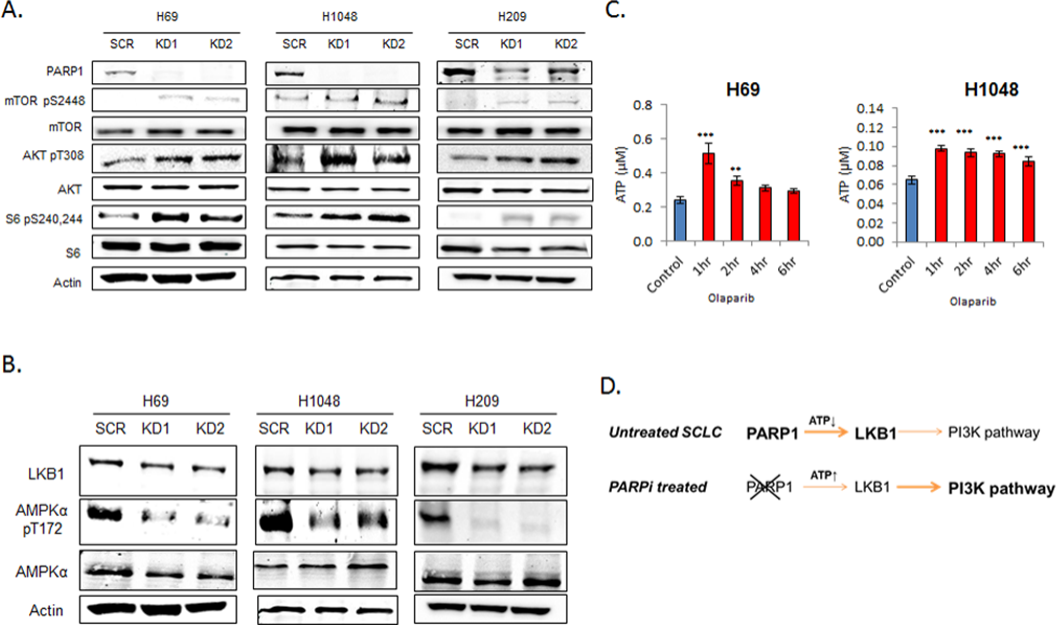
PARP inhibition inactivates the LKB1 pathway through increased ATP. (A) Lysates from PARP1 knockdown by shRNA in SCLC cell lines (H69, H1048, H209) results in increased activity of the PI3K/mTOR pathway relative to scramble (Scr) shRNA as determined by western blot. (B) *PARP1* knockdown shRNA cells (KD1 and KD2) had lower LKB1 and pAMPKα expression than scramble shRNA cells. (C) H69 and H1048 cells treated with olaparib showed increased [ATP] as measured by ATPlite assay. Data are presented as mean ± SEM; ** p<0.02, *** p<0.01. (D) A proposed model of PI3K pathway activation following PARP inhibition.

PARP inhibitors work through both the catalytic inhibition of PARP and a phenomena called PARP-DNA trapping where PARP is trapped at the site of a double stranded break (DSB) preventing that DSB from being repaired and causing direct cytotoxicity [26]. Therefore, to look at the effect of PARP catalytic inhibition specifically, we knocked down the *PARP1* gene by lentiviral shRNA transduction in a panel of SCLC cell lines including a representative cell line from the in vitro pharmacokinetic analysis and cell lines used in the animal studies (described below). PARP knockdown allowed us to not only look directly at the catalytic inhibition of PARP1, but to also avoid potential off-target effects of pharmacologic inhibitors. As shown in Fig 2A, this knockdown significantly reduced PARP1 expression compared to scramble shRNA (control). Further western blot analysis of *PARP1* shRNA knockdown cell lines showed increased mTOR, AKT, and S6 activity relative to scramble shRNA control in all cell lines tested, despite H69 and H1048 having activating mutations in *PIK3CA*. We confirmed our observations in H69 knockdown cells by using a limited RPPA panel analyzing selected proteins (S2 Fig). In this analysis, the second and third most significant differences between scramble- and *PARP1* shRNA–transduced cells were decreased PARP1 and increased p-S6 (of 35 hits in *PARP1* KD1 and 52 in KD2 at FDR≤0.05). The observed increased in PI3K/mTOR signaling with short-term pharmacological and long-term genetic loss of PARP1 function reinforces the notion that PI3K/mTOR activation is an effect that is related to the catalytic inhibition of PARP rather than PARP-DNA trapping. The novel observation that PARP inhibition or knockdown activates the PI3K/mTOR pathway in SCLC (Fig 1) is consistent with our previously published report that this pathway is the strongest marker of baseline resistance to PARP inhibition [5].

Further analysis of PARP1 activity on the LKB1 pathway in SCLC cell lines showed that LKB1 expression and phosphorylation of AMPKα were both lower in *PARP1* knockdown cells than in cells treated with scramble shRNA (Fig 2B).

PARP catalyzes the polymerization of ADP-ribose from NAD+ to attach poly ADP-ribose (PAR) to donor proteins in a process called PARylation [27]. As PARylation by PARP is an ATP-intensive event, we hypothesized that PARP inhibition results in increase of available ATP, which in turn results in decreased LKB1 pathway activity and therefore a loss of PI3K/mTOR suppression. Using a luminescence-based assay, we measured global ATP levels in SCLC cells before and after treatment with a PARP inhibitor. As shown in Fig 2C, ATP concentrations were increased following treatment with olaparib (1μM, p<0.001 at 1 hour). The proposed mechanism for how PARP inhibition can stimulate the PI3K/mTOR pathway via increased ATP availability and suppression of the LKB1 pathway is shown as Fig 2D. These observations concur with a recent report that PARP1-mediated ionizing radiation–induced autophagy occurs through activation of the LKB1/AMPK/mTOR pathway [28] as well as reports showing that the activation of PARP1 following alkylating DNA damage activates the LKB1 pathway to suppress PI3K signaling via the depletion of ATP–an effect that is lost following PARP1 inhibition [29, 30].

### Talazoparib and a PI3K inhibitor combine additively in SCLC

**Fig 3:**
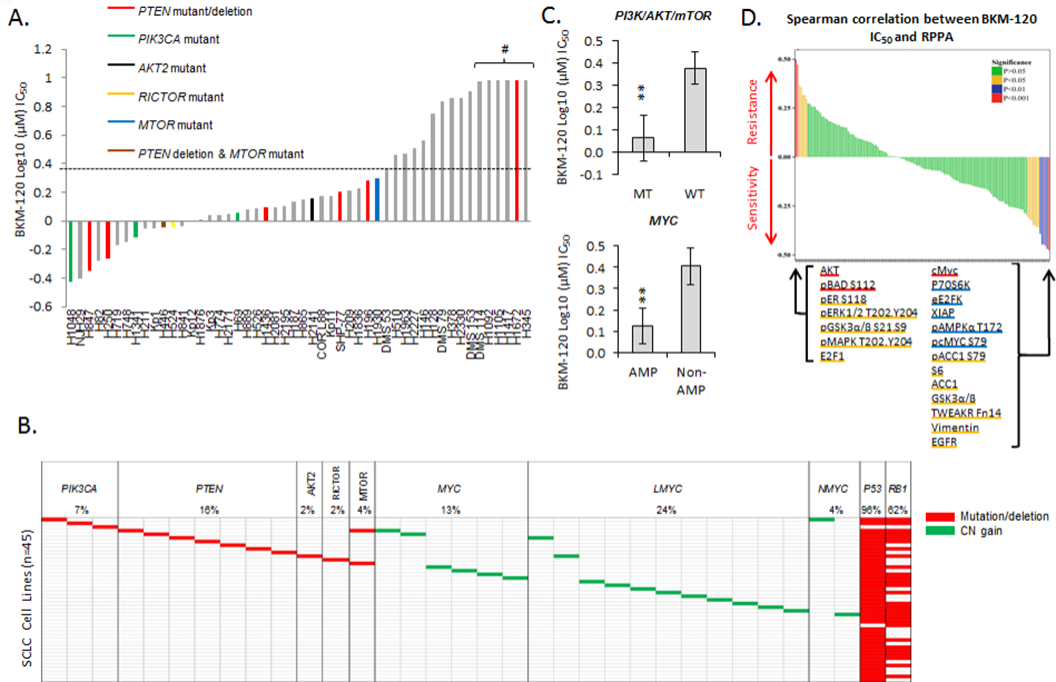
BKM-120 inhibits SCLC growth *in vitro*. (A) Proliferation assays showed a range of sensitivities to BKM-120 across the SCLC cell line panel. Cell lines with *PTEN* mutations/deletions are colored red and those with *PIK3CA* mutations green; clinical C_max_ is indicated with a dashed horizontal line. # indicates IC_50_ not reached at doses tested. (B) Summary of *PI3K/mTOR* mutation and *MYC* amplification status of the cell lines (see S3 Fig for cell line names). (C) Cell lines with either a mutation in the PI3K/mTOR pathway (MT) or a *MYC* amplification (AMP) were more sensitive to BKM-120 than cells without a mutation or amplification (WT and NON-AMP; ** p<0.02.). (D) Biomarker discovery using the RPPA profiles of 47 SCLC cell lines correlated protein expression with BKM-120 IC_50_ to identify markers predictive of response. Proliferation data are presented as mean ± SEM.

Expanding on our previous observations that 1) sensitivity to PARP inhibition is related inversely to PI3K/mTOR pathway activity [5] and 2) PARP inhibition increased PI3K/mTOR signaling, we tested the anti-proliferation efficacy of a PI3Kα inhibitor (BKM-120) in our panel of 50 SCLC cell lines. BKM-120 (a pan-class-1 P110α/β/γ/δ inhibitor) was chosen because it was used in a study of a PARP/PI3K inhibitor combination in breast/ovarian cancer (ClinicalTrial.gov Identifier: NCT01623349) and because another P110-specific inhibitor (PIK75) has been shown to have single-agent activity in some SCLC cell lines [12]. As shown in Fig 3A, IC_50_ was reached in a majority of cell lines tested (44/50), 35 of those at a clinically achievable dose less than the reported day 1 C_max_ of 2.3μM [31].

To determine the role that PI3K/mTOR pathway mutations may play in sensitivity to BKM-120, we identified cell lines with such mutations. Cells with *PIK3CA* mutations (n = 3, indicated in green in Fig 3A) were associated with lower IC_50_ values, indicating, as would be expected, that cells that are more dependent on PI3K/mTOR signaling for survival are more sensitive to a PI3K inhibitor. We also identified *AKT2*, *RICTOR*, and *MTOR* mutations in cell lines that were sensitive to BKM-120. Cells with *PTEN* alterations (n = 7, indicated in red in Fig 3A) were distributed across the range of sensitivities, implying that *PTEN* alterations do not correlate with sensitivity. The frequencies of mutations in these five genes (summarized in Fig 3B and S3 Fig) are similar to those published in a recent study of SCLC biopsy specimens [10]. When grouped together, cell lines with PI3K/mTOR pathway mutations had significantly lower IC_50_ values to BKM-120 than non-mutated cells (Fig 3C, p = 0.01).

To identify potential proteomic markers of response to BKM-120, we analyzed the correlation between IC_50_ to BKM-120 and basal expression levels of 146 total or phosphorylated proteins by RPPA. As shown in Fig 3D, Spearman correlation identified cMyc protein expression as the top marker of sensitivity to BKM-120 (p<0.001); other significant (p<0.05) markers of sensitivity included p-cMyc, p-AMPKα, and p-ACC1. Further analysis of the BKM-120 sensitivity data revealed that cell lines with a known *MYC* amplification had a significantly lower IC_50_ (p = 0.01, Fig 3C), validating the proteomic marker. Among the strongest markers of resistance to BKM-120 were proteins implicating the MAPK/ERK pathway (pERK1/2 (T202,Y204) Rho = 0.358, p = 0.014; pGSK3α/β (S21,9) Rho = 0.318, p = 0.032; pMAPK (T202,Y204) Rho = 0.317, p = 0.032). Activation of the MAPK/ERK pathway following inhibition of PI3K has been observed in a number of cancers [32], including NSCLC [33] and breast cancer [32, 34]; this effect may be mediated through loss of AKT’s inhibitory effect on Raf [35]. Beyond the MAPK/ERK pathway, the strongest markers of resistance to BKM-120 were AKT and p-BAD (p<0.001) Interestingly, BKM-120 IC_50_ values did not show a strong correlation with our previously published PI3K score [5] (Rho = 0.101, p = 0.51), which is consistent with published observations that sensitivity to pictilisib (GDC0941, a PI3Kα/δ inhibitor) showed no correlation with the PI3K score in a panel of 60 head and neck squamous cell carcinoma cell lines [36]. These observations suggest that the activation of MEK (or other compensatory/escape pathways) may be more important than basal PI3K pathway activity in determining sensitivity to PI3K inhibition.

**Fig 4:**
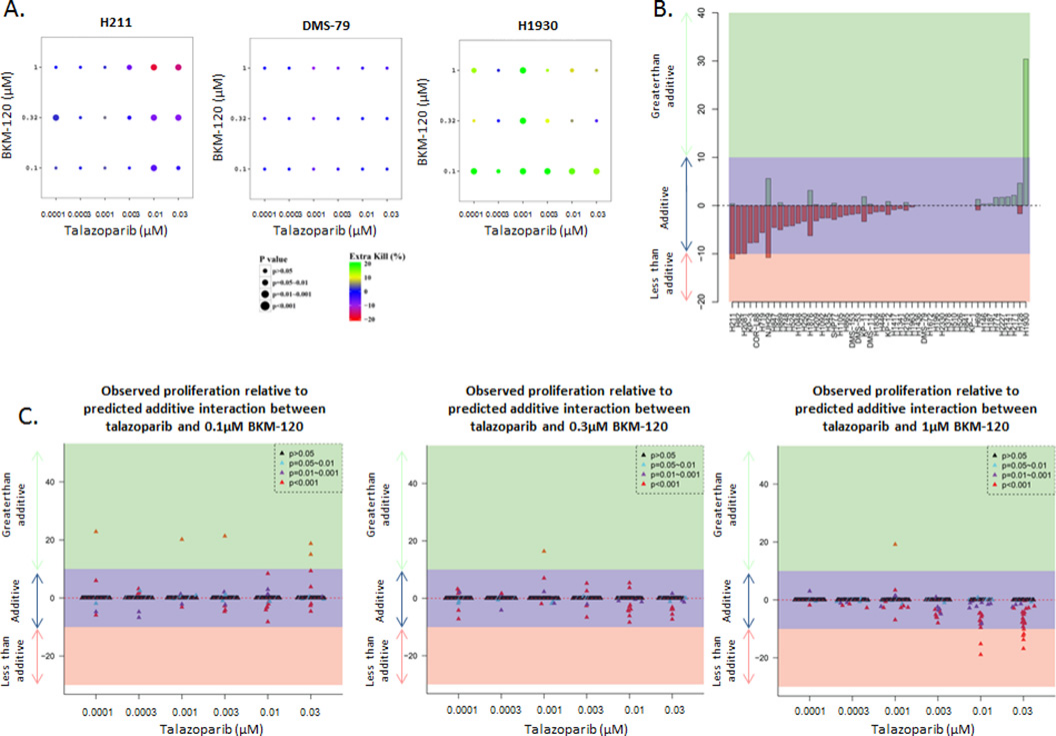
Talazoparib and BKM-120 act additively to inhibit SCLC growth *in vitro*. (A) Proliferation assays using clinically achievable doses of talazoparib (6 doses, range 0.1–30 nM) and BKM-120 (3 doses, range 0.1–1 μM) showed an additive interaction in most of the 50 SCLC cell lines tested. Examples show additive responses (H211, DMS-79), and a greater-than-additive response (H1930). (B) Degree (percentage) of inhibition above (green) or below (pink) the predicted additive effect (purple) for each cell line across all clinically achievable doses. (C) Observed proliferation relative to predicted additive effect at individual doses of BKM-120.

The combination of PARP and PI3K inhibitors has been reported to be highly active in preclinical models (patient-derived xenografts and a *Brca1/Trp53* knockout spontaneous genetically engineered mouse model) of breast cancer [13, 14]. Based on these results, a clinical trial testing the combination of the PARP inhibitor olaparib and a PI3K inhibitor (pan-P110 BKM-120 or P110α specific BYL719) was initiated for breast and ovarian cancer (NCT01623349). Preliminary data demonstrates the tolerability of the combination [37] and clinical benefit at all dose levels in at least the olaparib + BKM-120 cohort [38]. We therefore tested the effects of a combination of talazoparib (PARP inhibitor) and BKM-120 (PI3K inhibitor) at clinically achievable doses in a panel of SCLC cell lines with a range of sensitivities (sensitive, intermediate and resistant) to both BKM-120 and talazoparib (sensitivity to talazoparib shown in S4 Fig). Six doses of talazoparib (range 0.1–30 nM) and three doses of BKM-120 (range 0.1–1 μM) were tested in each cell line. At each dose level, we compared the effect on proliferation with the predicted additive effect. Proliferation rates that were within 10% of the predicted additive effect where considered “additive,” while whose with proliferation rates more than 10% above or below the predicted additive effect were considered “less than additive” or “greater than additive” (Fig 4A). As an example, Fig 4A shows representative cell lines with an “additive” response (H211, DMS-79) or a “greater than additive” response (H1930). Using this approach, we demonstrated an additive interaction in 49 of the 50 SCLC cell lines tested.

To take a global view of the interaction between the two drugs for each cell line across all doses, we performed a further analysis that combined the degree (percentage) of inhibition above or below the predicted additive effect from all possible combinations. Using the area under curve (volume), we compared the observed and predicted additive values (as a percentage relative to predicted) at for each combination (with a unit of μM^2^%) the positive and negative values were then separately summed for each cell line (Fig 4B). For all but three cell lines, the overall effect of the talazoparib + BKM120 combination was within a volume of ± 10% which fell within the predicted additive effect, further supporting the conclusion that these drugs were primarily additive *in vitro*.

An analysis looking at each dose of BKM-120 individually (Fig 4C) showed that the vast majority of cell lines had greater-than-additive responses (>10% greater than predicted additive response as described for Fig 4A) were at the lowest dose of BKM-120 (at which BKM-120 would have the fewest off-target effects). At higher doses of BKM-120, fewer greater-than-additive responses were seen, with some cell lines showing a less-than-additive response at the highest doses of BKM-120, in large part because, at these doses, the cells are very sensitive to single-agent treatment.

**Fig 5:**
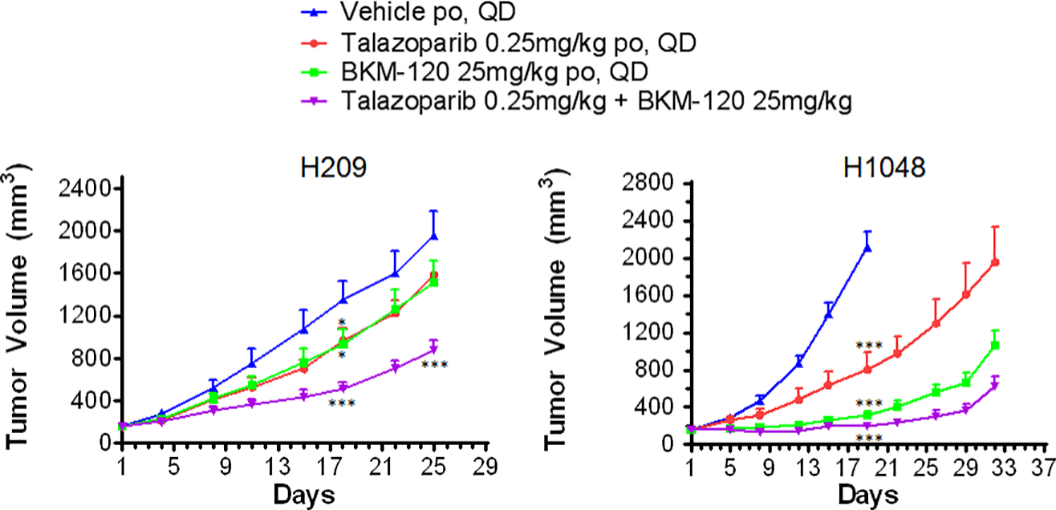
Talazoparib and BKM-120 interact synergistically to inhibit SCLC growth *in vivo*. Animals bearing an H209 or H1048 xenograft on their flank were treated with talazoparib, BKM-120, or the combination once tumors reached an average volume of 150mm^3^; effects of the treatments on tumor growth are shown. Each treatment delayed tumor growth, but the combination treatment had a greater effect than the predicted additive effect of each agent alone at day 18 and 25 in the H209 model (Day 18: predicted ΔT/ΔC = 0.33, observed = 0.30; Day 25: predicted ΔT/ΔC = 0.55, observed = 0.40). Data are presented as mean ± SEM, *p<0.05, ***p<0.01.

Having observed an additive effect in cell lines, we further tested the combination of talazoparib + BKM-120 in two xenograft models of SCLC (H209 and H1048). As shown in Fig 5, the combination treatment was more effective than either treatment alone in slowing xenograft development (H209: p = 0.002 vs talazoparib, p = 0.008 vs BKM-120; H1048: p = 0.002 vs talazoparib, p = 0.008 vs BKM-120, both at day 18). Furthermore, we observed no significantly increased toxicity or weight loss in the animals that received the combination (S5 Fig). In the H209 model, the effect of the combination at day 25 of treatment (ΔT/ΔC = 0.4) was greater than the predicted additive value (ΔT/ΔC = 0.55) of either agent alone (talazoparib ΔT/ΔC = 0.79, BKM-120 ΔT/ΔC = 0.75), suggesting a synergistic effect *in vivo*. The H1048 cell line has an activating mutation in *PIK3CA* which, as expected from it being the most sensitive cell line when tested *in vitro* (Fig 2A), increases sensitivity to the PI3K inhibitor *in vivo* (BKM-120 ΔT/ΔC = 0.08 at day 19). As a consequence of this model being extremely sensitive to PI3K inhibition *in vivo*, the additional effect seen with the combination treatment is limited (ΔT/ΔC = 0.02). These observations suggest that combining PARP and PI3K inhibitors could be of therapeutic benefit beyond patients with *PIK3CA* mutant disease.

### Talazoparib and BKM-120 have independent and complementary mechanisms of action

**Fig 6:**
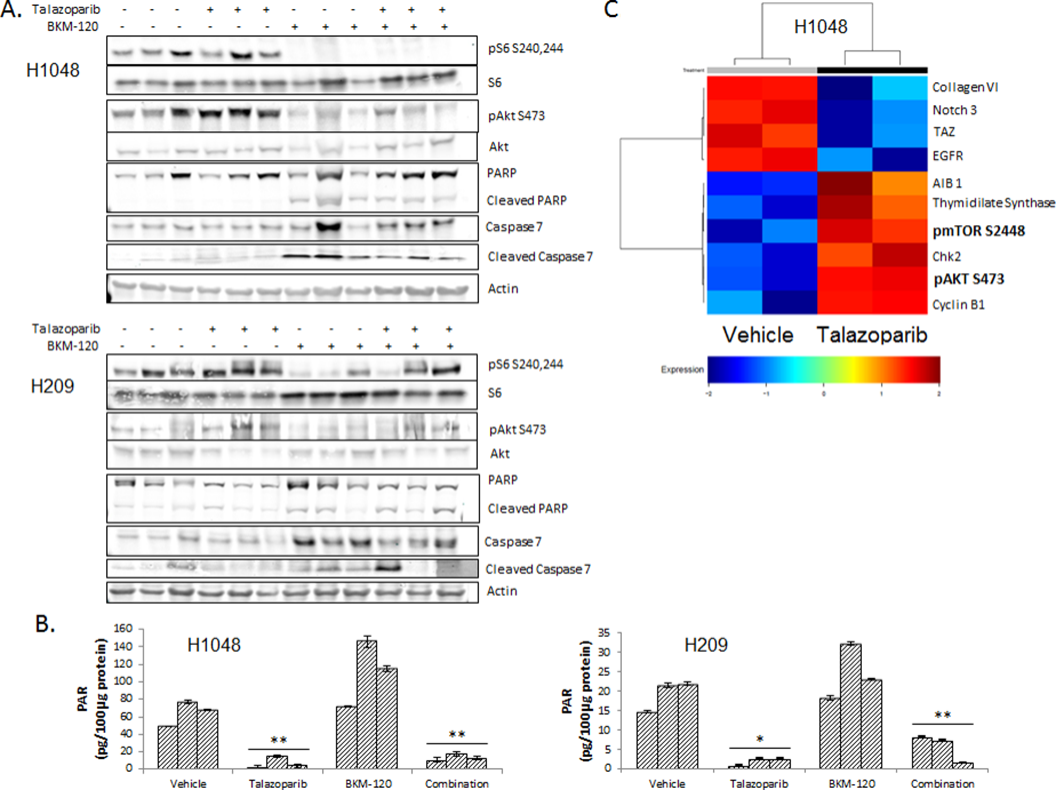
Talazoparib and BKM-120 have different, complementary modes of action. (A) Lysates prepared from H1048 and H209 xenografts (3 per treatment group) harvested 2 hours after treatment on day 3 showed increased p-AKT (S473) and p-S6 (S240,244) with talazoparib treatment alone but reduced signaling with BKM-120 treatment alone or the combination (quantification of western blots shown in S6 and S7 Figs). (B) PARP activity, as measured by PAR levels, was decreased in both xenograft models following talazoparib treatment. (C) H1048 xenograft lysates treated with talazoparib and analyzed by RPPA in an independent experiment show increased p-mTOR (S2448) and p-AKT (S473) following treatment.

Having observed increased PI3K signaling following PARP inhibition *in vitro*, and a greater-than-additive effect with the talazoparib + BKM-120 combination *in vivo*, we assayed protein expression by western blot in lysates from H1048 and H209 flank xenografts treated with talazoparib, BKM-120, or the combination. As shown in Fig 6A, short-term talazoparib treatment (3 days) of H1048 resulted in increased expression of activated AKT (p-AKT S473, p = 0.04, quantification shown in S6 Fig) with no change in total AKT. As expected from our previous studies [5], talazoparib treatment decreased PAR levels, indicating strong inhibition of PARP catalytic activity (Fig 6B) without affecting total PARP1 expression (Fig 6A). The observed increase in PI3K/mTOR signaling was validated in H1048 xenograft lysates treated with talazoparib in an independent experiment (Fig 6C) where increased p-mTOR (S2448) and p-AKT (S473) were among the most significant changes (p = 0.023 and p = 0.008, respectively). Short-term treatment of H209 xenografts resulted in a modest increase in p-AKT (p = 0.2, Fig 6A, quantified in S6 Fig) with no change in total AKT, and also decreased PARP activity (Fig 6B). Unlike *in vitro*, we did not see significant changes in LKB1 pathway levels, however tumor lysates were collected on the third day of treatment whereas *in vitro* experiments used a 24 hour time point which may account for the less significant changes seen in the tumors. The third day of treatment may also no longer reflect immediate changes in intercellular signaling and could be impacted by changes such as increased apoptosis.

Conversely, BKM-120 treatment in H1048 xenografts reduced AKT activity (p = 0.04) and eliminated p-S6 expression (p = 0.008), as seen on western blots (Fig 6A, quantified in S5 Fig). Cleaved PARP was also increased following treatment with BKM-120 (p = 0.01), suggesting that, unlike with the PARP inhibitor, which is believed to elicit a majority of its cytotoxic effect through PARP trapping [26], BKM-120 kills SCLC cells through apoptosis, which has been reported elsewhere as the primary mode of cell killing by BKM-120 [39]. Similar responses were seen in H209 xenografts: BKM-120 treatment reduced phosphorylation of S6 and AKT (p = 0.02 and p = 0.1, respectively), with a modest increase in cleaved PARP (p = 0.1; Fig 6A, quantified in S7 Fig). The less significant response of H209 xenografts to BKM-120 likely reflects H209’s lack of known mutations in the PI3K/mTOR pathway (H209 also was less sensitive to BKM-120 *in vitro* than H1048).

In H1048 tumors treated with the combination of talazoparib + BKM-120, p-AKT (p = 0.04) and PARP activity (p = 0.004) were decreased, p-S6 was eliminated (p = 0.008), and cleaved PARP was increased (p = 0.01). Decreased PARP activity and increased cleaved PARP expression suggest that the two inhibitors inhibit proliferation by different, complementary mechanisms (cytotoxic PARP trapping versus apoptosis). The elevated level of cleaved PARP in the combination treatment group was notable, suggesting that the apoptotic functions of PARP may continue in the presence of a PARP inhibitor. Indeed, a second marker of apoptosis cleaved caspase-7 expression was increased in both the BKM-120 and combination treatment groups (p = 0.01 and p = 0.001, respectively), with a corresponding increase in caspase-7 in the combination treatment group (p = 0.02). As with the individual treatments, H209 xenografts responded similarly to the combination, with increases in cleaved PARP (p = 0.02) and cleaved caspase-7 (p = 0.3).

## Discussion

PARP1 is overexpressed in many human cancers, particularly SCLC [4], and is being developed as a target in the treatment of a number of cancers including those of the ovary, breast, prostate and lung. The mechanism of action of PARP inhibitors in SCLC appears to be different from other cancers such as ovarian and breast where sensitivity is in large part driven by a synthetic lethality with *BRCA* mutations or other mutations in genes regulating homologous recombination [40, 41]. BRCA deficient tumors have been shown to become resistant to PARP inhibition through a number of mechanisms including genetic reversion, hypomorphic activity, rewiring of the DNA damage response, and increased dug efflux [42]. In SCLC however where *BRCA* mutations are rare (1–3.5% [9, 43]), we have previously shown that resistance to PARP inhibition to be correlated to activation of the PI3K/mTOR pathway [5]. In this study we found that the inhibition of PARP1 resulted in increased PI3K/mTOR signaling, suggesting that SCLC may attempt to escape PARP inhibition by upregulation of the PI3K/mTOR pathway. We further observed that long-term suppression of PARP1 expression by gene knockdown also resulted in increased PI3K/mTOR signaling. The observations made here that PARP inhibition increased PI3K/mTOR signaling, in combination with our previous report that SCLC cell lines with higher PI3K/mTOR activity are less sensitive to PARP inhibitor talazoparib, suggest that this pathway is a both a marker of inherent resistance and a potential mechanism of acquired resistance to PARP inhibition.

Further interrogation of our RRPA data comparing PARPi treated and untreated cells revealed a decrease in LKB1 signaling. The LKB1 pathway is a stress response pathway that is activated in response to stresses such as ATP depletion. Our data suggest PARPi increases the availability of ATP and reduces LKB1 signaling driving the activation of the PI3K/mTOR patway. A similar effect is seen following PARP1 knockdown, albeit with a greater effect upon AMPKα activation than LKB1 expression. Previous reports have shown that in addition to LKB1, AMPKα activity can be modulated by the loss of LKB1’s binding partners MO25 and STRAD (that LKB1 must complex with to activate AMPKα), and also the loss of the promotion of phosphorylation by ATP/ADP, inhibition of dephosporylation and promotion of the allosteric activation of phosphorylated AMPKα [44]. Further, the phosphatases for AMPKα (which are not definitively known [44] but may include PP2A [45] and αSNAP [46]) are not included in the RPPA panel and were not included in these analyses. We therefore hypothesize that in the longer duration knockdown experiments, as opposed to the 24 hour PARPi experiments, the inactivation of AMPKα is not only being driven by reduced LKB1 expression. However, these observations raise the intriguing possibility that AMPKα targeting via metformin or other drugs in addition to PARPi could achieve a similar effect as the PARP/PI3K inhibitor combination, but with the potential for fewer adverse side-effects.

On the basis of these findings, we tested combinations of a PARP inhibitor and a PI3K inhibitor to determine if this would inhibit activation of the PI3K/AKT pathway and thereby provide greater anticancer effect than PARP inhibition alone. Initial testing of the PI3K inhibitor BKM-120 as a single agent found significant activity in over half of the SCLC cell lines tested, ranging from very sensitive (e.g., H1048) to resistant (e.g., H345). Subsequent testing of talazoparib + BKM-120 revealed that the combination was predominantly additive *in vitro*. We had anticipated that the talazoparib + BKM-120 would have a greater than additive effect *in vitro*, suggesting that while we had good reason to choose BKM-120 it may not be the best agent to use in combination with talazoparib. BKM-120 is not the most potent inhibitor of PI3K. BYL719 (also included on trial NCT01623349), for example, has a lower IC_50_ to P110α (5nm vs 52nM [47, 48]) and BKM-120 also inhibits other members of the P110 family. Another clinical trial (NCT02338622) combines olaparib with the pan-AKT inhibitor AZD5363 (IC_50_ 1-8nM [49]) which might be a better target for combination treatments. When tested *in vivo*, however, the combination of talazoparib + BKM-120 delayed tumor growth to a significantly greater degree than either talazoparib or BKM-120 alone, supporting further investigation of a PARP-PI3K inhibitor combination in SCLC clinical trials. A similar sensitization to PARP inhibition by BKM-120 was seen in homologous recombination proficient breast cancer, where PI3K inhibition lead to a decrease in BRAC1/2 expression [13].

We examined biomarkers that were associated with sensitivity to both BKM-120 and the talazoparib + BKM-120 combination to identify potential markers of response that could eventually be applied to patient selection. Interestingly, in clinical samples, mutations in the PI3K/mTOR pathway do not appear to overlap with amplifications in the *MYC* family, another relatively common event in SCLC (~16% of cases) that has been reported in a human mammary epithelial cell (HMEC) model of acquired resistance to BEZ235, which targets PI3K and ATR [50]. *MYC*-amplified and Myc-overexpressing cell lines both showed relatively higher sensitivity to BKM-120, indicating that patients with a *MYC*-amplified tumor may do well with such treatment despite previous reports of *MYC* amplification in a HMEC model of acquired resistance to BEZ235; this disparity may reflect the fact that BEZ235 exerts it anticancer effects primarily through ATR inhibition [51]. We previously observed that cMyc-overexpressing cell lines tended to be more sensitive than other cell lines to talazoparib (R = -0.262, p = 0.5; S8 Fig). These observations suggest that Myc-overexpressing/amplified SCLC may respond well to the combination of PARP and PI3K inhibition, but this could not be tested in these experiments because of the limited range of response seen *in vitro*. Further animal studies using a greater number of models may cast light upon this possibility.

Single-agent PI3K inhibition was less effective against cells with a more active MEK/ERK pathway. From our previous comparison of SCLC and NSCLC [4], we would consider SCLC with more active MEK/ERK signaling to be closer to the NSCLC end of the spectrum of SCLC, perhaps explaining why these cells are less sensitive to BKM-120. The similarities between baseline sensitivity to both PARP and PI3K inhibition, where MEK/ERK signaling predicts resistance to both (Fig 3D and S8 Fig), suggest that MEK/ERK activation is a potential mechanism of resistance to the combination treatment. The development of models with dual acquired resistance to PARP/PI3K inhibitors may be informative in addressing this possibility.

The combination of PARP and PI3K inhibition *in vivo* reduced tumor growth greater than the predicted additive effect of the single agent treatments. Subsequent proteomic analysis indicates that the independent mechanisms of cell death following PARP and PI3K inhibition (cytotoxicity vs induction of apoptosis) continue independently in the combination treatment. The observed greater than additive effect is likely a consequence of the PI3K inhibitor reversing/preventing innate and acquired resistance to the PARP in addition to its own anti-cancer effect.

Finally, the observation that talazoparib combines more than additively with BKM-120 in animal models of SCLC provides a strong rationale for the rapid advancement of clinical studies co-targeting PARP and PI3K (or AKT) in these cancers. This is especially true in light of recently opened trials of such combinations in breast and ovarian cancers (NCT01623349, NCT02338622).

## Supporting Information

S1 Fig Talazoparib treatment increases PI3K/mTOR signaling *in vitro*. Western blot analysis of olaparib and talazoparib treated H69 cells. Lysates collect at 24 hours treatment.
(TIF)

S2 Fig PARP knockdown decreases PARP and increases PI3K/mTOR signaling *in vitro*. RPPA analysis of lysates from PARP knockdown by shRNA in H69 shows decreased PARP and increased phosphorylated S6 as compared to scramble shRNA.
(TIF)

S3 Fig Mutations in PI3K/mTOR and myc amplification in SCLC cell lines. Known PI3K/mTOR mutations and myc amplifications in the SCLC cell line panel.
(TIF)

S4 Fig Talazoparib IC50 values in SCLC. IC50 values calculated from 5-day proliferation assays. # indicates IC50 not reached at maximum dose of 1μM.
(TIF)

S5 Fig Talazoparib combined with BKM-120 is well tolerated in animal models. Animal body weight is unaffected by either agent alone or in combination in both animal models tested.
(TIF)

S6 Fig Quantification of western blots using lysates from the 1048 animal model. Quantification of western blots reveals significant changes in protein expression (* p<0.05; ** p<0.02).
(TIF)

S7 Fig Quantification of western blots using lysates from the H209 animal model. Quantification of western blots reveals significant changes in protein expression (* p<0.05; ** p<0.02).
(TIF)

S8 Fig cMyc, pMEK, and pERK expression correlate weakly with talazoparib concentration. (A) Median inhibitory concentrations of talazoparib in 10-day assays (Cardnell *et al* 2013) correlated with cMyc expression (by RPPA) shows that cell lines with higher cMyc tended to be more sensitive to talazoparib. (B,C) Cell lines with greater activation of MEK (B) and ERK (C) tended to be less sensitive to talazoparib.
(TIF)
