# Supplementary figures and images for: Activation of the PI3K/mTOR Pathway following PARP Inhibition in Small Cell Lung Cancer
Source: PLoS One. 2016 Apr 7;11(4):e0152584. doi: 10.1371/journal.pone.0152584 (PMC4824499; doi:10.1371/journal.pone.0152584)

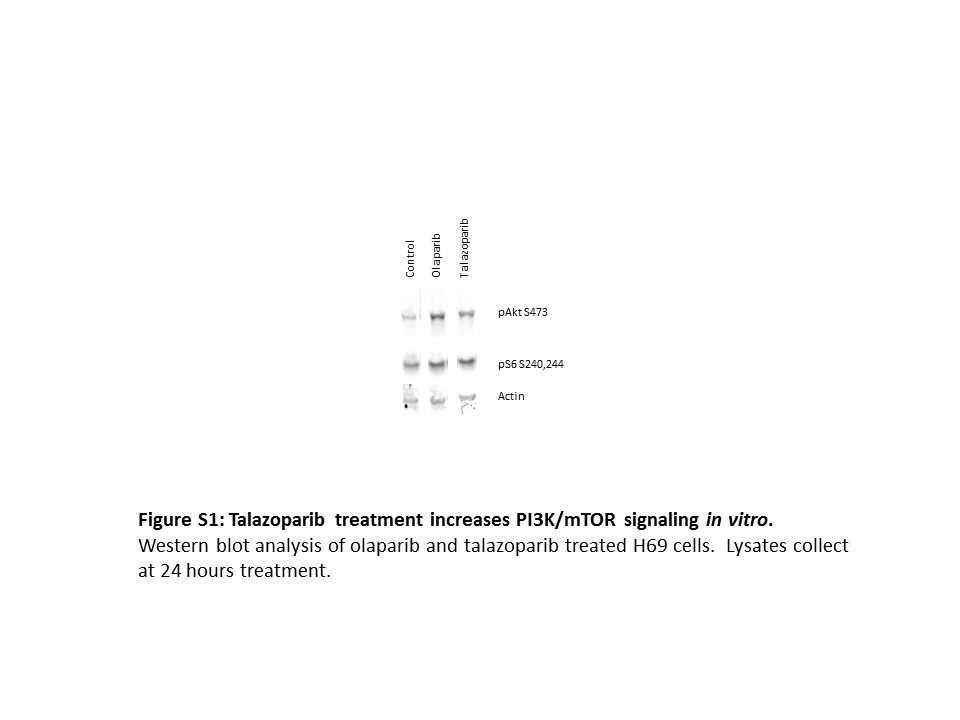

Supplement: S1 Fig — Western blot analysis of olaparib and talazoparib treated H69 cells. Lysates collect at 24 hours treatment. (TIF) [file pone.0152584.s001.tif]

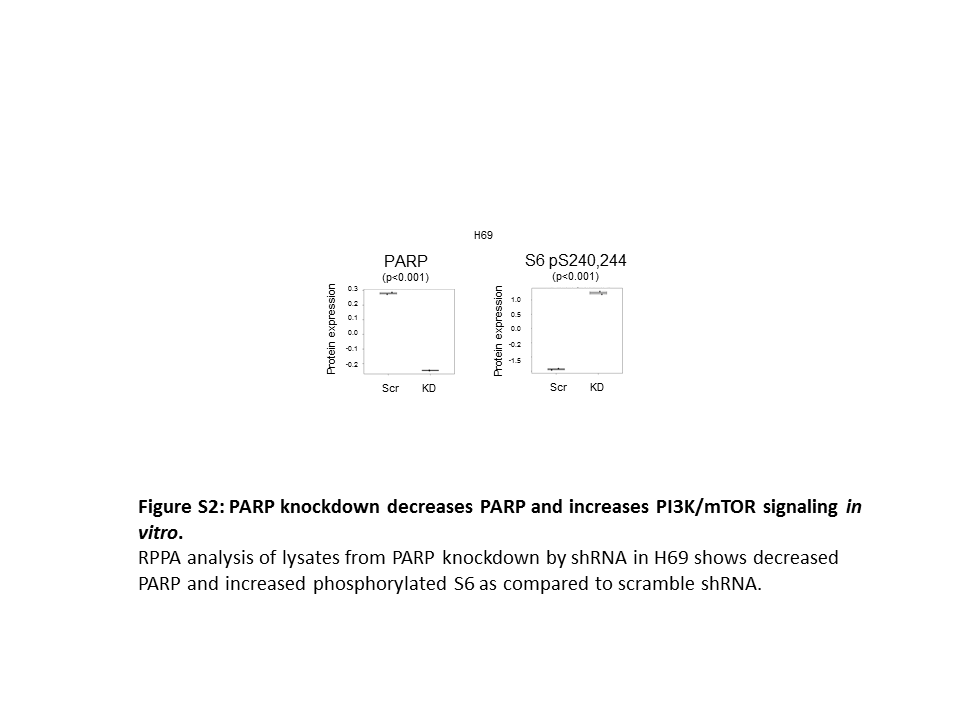

Supplement: S2 Fig — RPPA analysis of lysates from PARP knockdown by shRNA in H69 shows decreased PARP and increased phosphorylated S6 as compared to scramble shRNA. (TIF) [file pone.0152584.s002.tif]

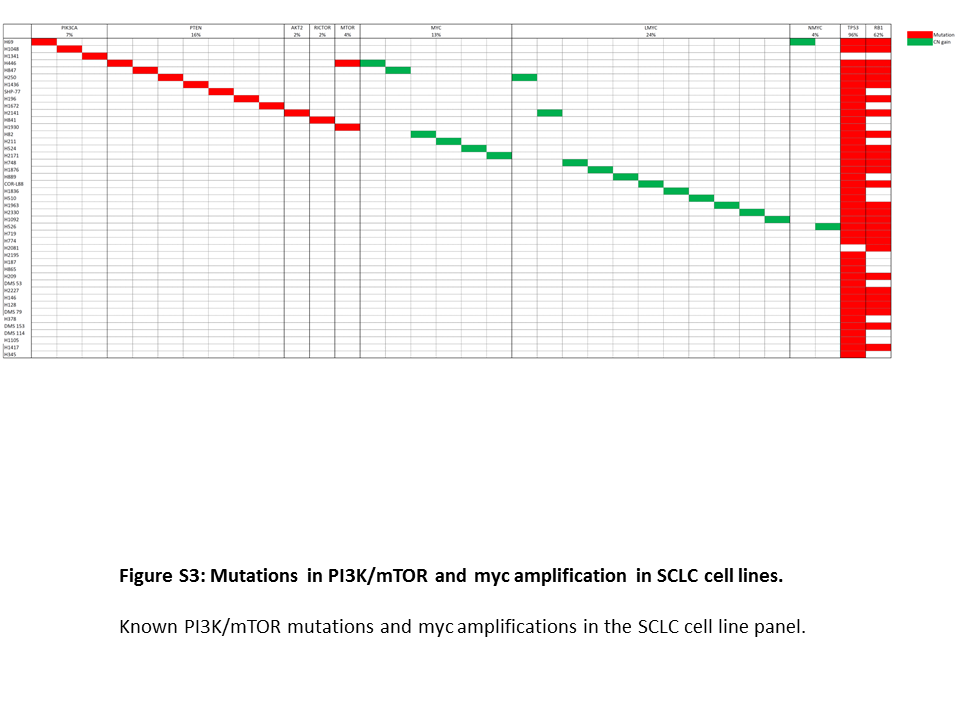

Supplement: S3 Fig — Known PI3K/mTOR mutations and myc amplifications in the SCLC cell line panel. (TIF) [file pone.0152584.s003.tif]

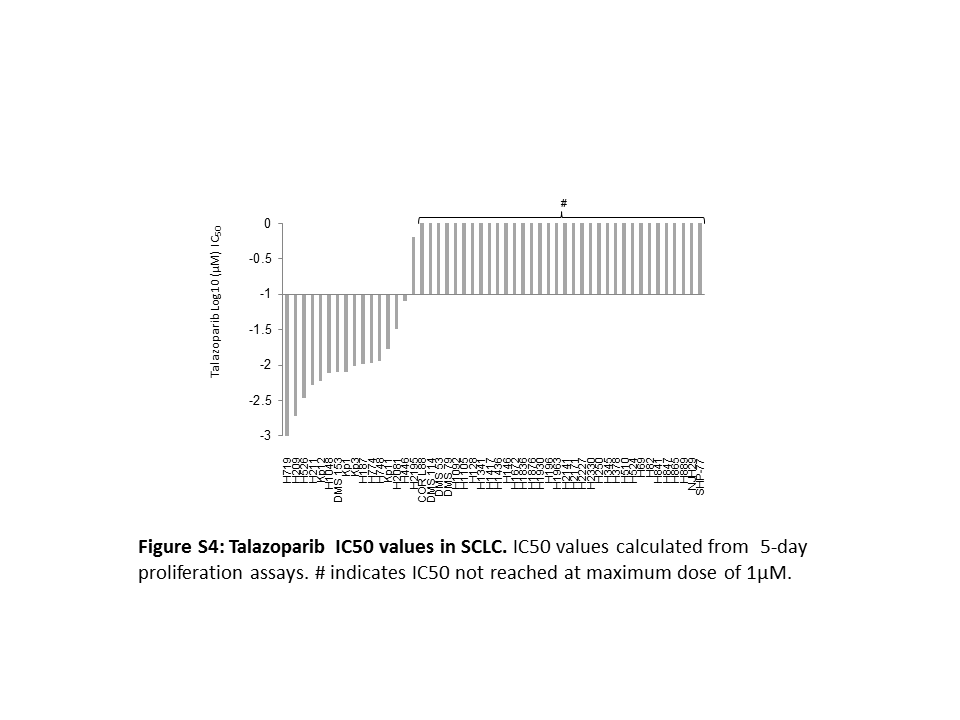

Supplement: S4 Fig — IC50 values calculated from 5-day proliferation assays. # indicates IC50 not reached at maximum dose of 1μM. (TIF) [file pone.0152584.s004.tif]

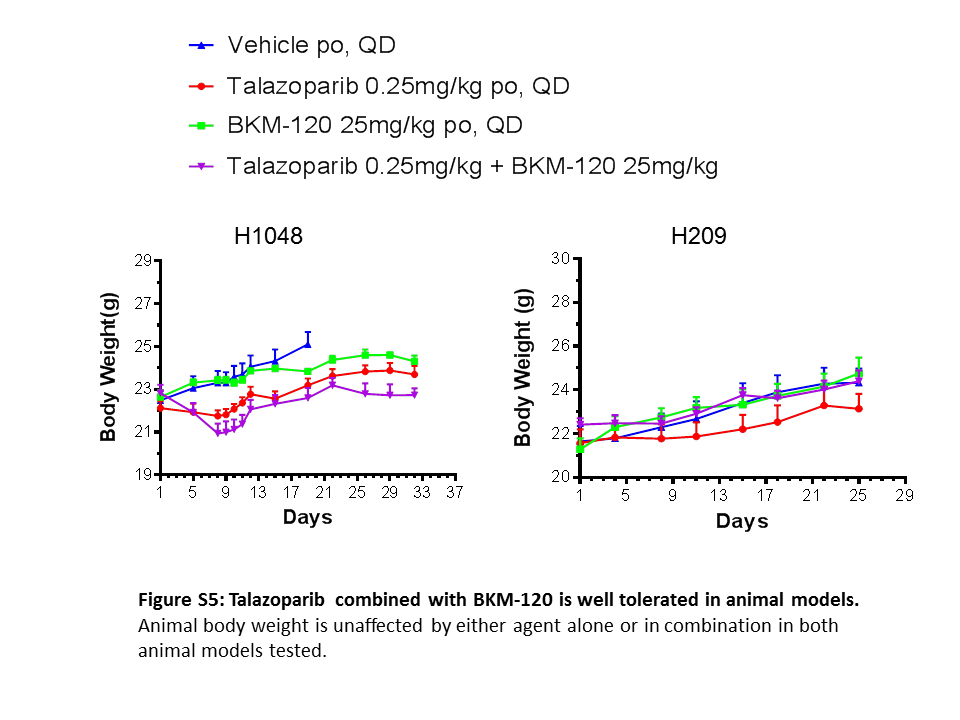

Supplement: S5 Fig — Animal body weight is unaffected by either agent alone or in combination in both animal models tested. (TIF) [file pone.0152584.s005.tif]

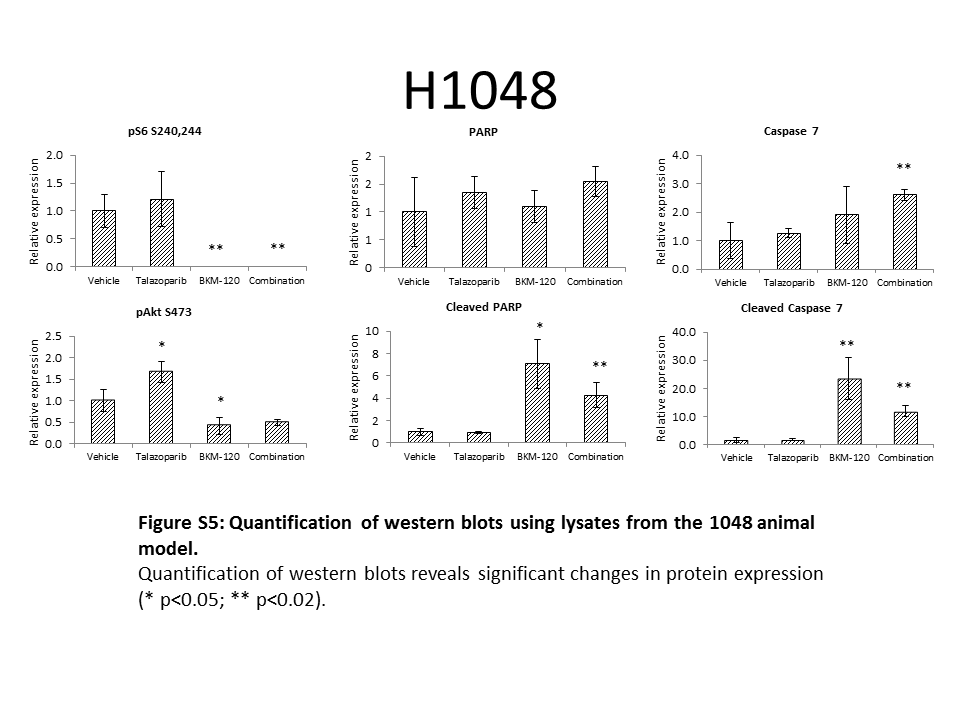

Supplement: S6 Fig — Quantification of western blots reveals significant changes in protein expression (* p<0.05; ** p<0.02). (TIF) [file pone.0152584.s006.tif]

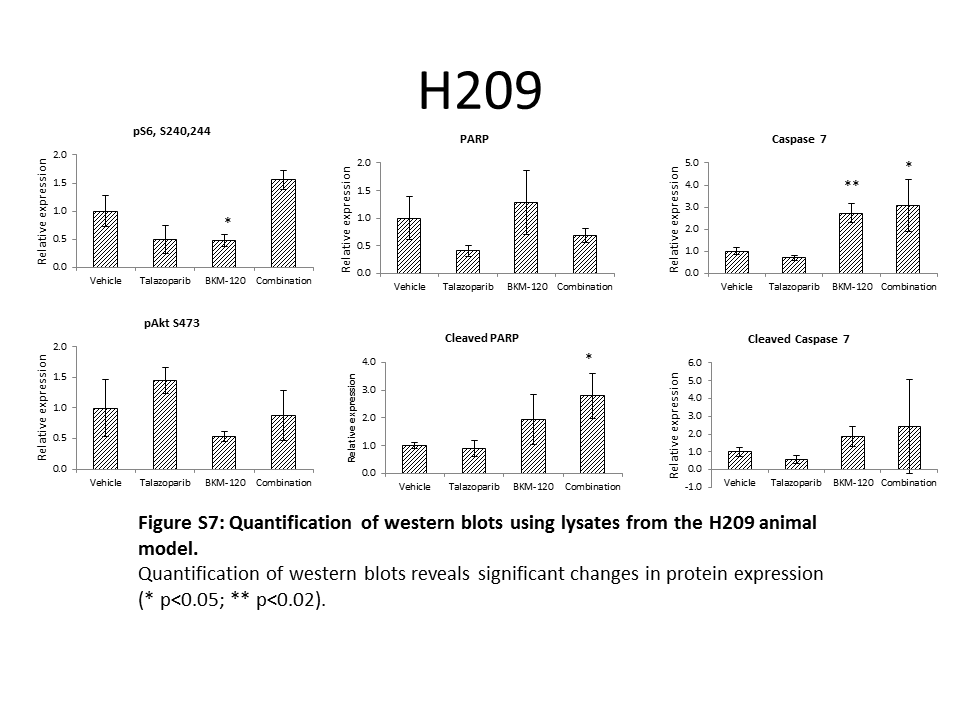

Supplement: S7 Fig — Quantification of western blots reveals significant changes in protein expression (* p<0.05; ** p<0.02). (TIF) [file pone.0152584.s007.tif]

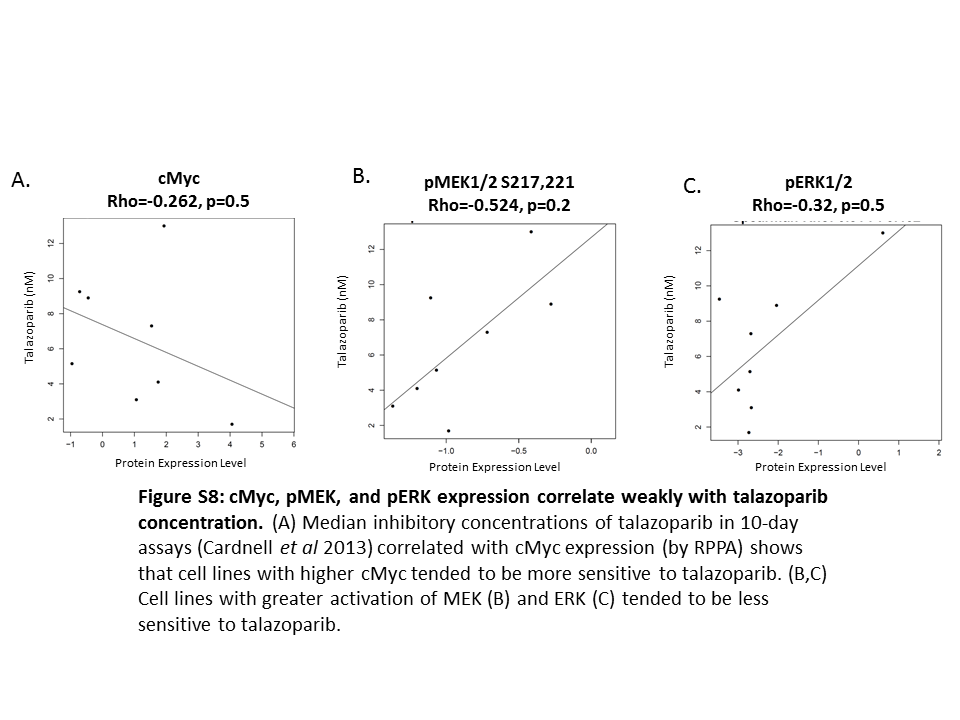

Supplement: S8 Fig — (A) Median inhibitory concentrations of talazoparib in 10-day assays (Cardnell et al 2013) correlated with cMyc expression (by RPPA) shows that cell lines with higher cMyc tended to be more sensitive to talazoparib. (B,C) Cell lines with greater activation of MEK (B) and ERK (C) tended to be less sensitive to talazoparib. (TIF) [file pone.0152584.s008.tif]
